# Selection and validation of a set of reliable reference genes for quantitative RT-PCR studies in the brain of the Cephalopod Mollusc *Octopus vulgaris*

**DOI:** 10.1186/1471-2199-10-70

**Published:** 2009-07-14

**Authors:** Maria Sirakov, Ilaria Zarrella, Marco Borra, Francesca Rizzo, Elio Biffali, Maria Ina Arnone, Graziano Fiorito

**Affiliations:** 1 Laboratorio di Neurobiologia, Stazione Zoologica Anton Dohrn, Villa Comunale, 80121 Napoli, Italy; 2 Molecular Biology Core Service, Stazione Zoologica Anton Dohrn, Villa Comunale, 80121 Napoli, Italy; 3 Cellular and Developmental Biology, Stazione Zoologica Anton Dohrn, Villa Comunale, 80121 Napoli, Italy; 4 Functional and Evolutionary Ecology, Stazione Zoologica Anton Dohrn, Villa Comunale, 80121 Napoli, Italy; 5 Ecole Normale Supérieure de Lyon, Institute of Functional Genomics of Lyon 69964 Lyon, Cedex 07, France

## Abstract

**Background:**

Quantitative real-time polymerase chain reaction (RT-qPCR) is valuable for studying the molecular events underlying physiological and behavioral phenomena. Normalization of real-time PCR data is critical for a reliable mRNA quantification. Here we identify reference genes to be utilized in RT-qPCR experiments to normalize and monitor the expression of target genes in the brain of the cephalopod mollusc *Octopus vulgaris*, an invertebrate. Such an approach is novel for this taxon and of advantage in future experiments given the complexity of the behavioral repertoire of this species when compared with its relatively simple neural organization.

**Results:**

We chose *16S*, and *18S *rRNA, *actB*, *EEF1A*, *tubA *and *ubi *as candidate reference genes (housekeeping genes, HKG). The expression of *16S *and *18S *was highly variable and did not meet the requirements of candidate HKG. The expression of the other genes was almost stable and uniform among samples. We analyzed the expression of HKG into two different set of animals using tissues taken from the central nervous system (brain parts) and mantle (here considered as control tissue) by BestKeeper, geNorm and NormFinder. We found that HKG expressions differed considerably with respect to brain area and octopus samples in an HKG-specific manner. However, when the mantle is treated as control tissue and the entire central nervous system is considered, NormFinder revealed *tubA *and *ubi *as the most suitable HKG pair. These two genes were utilized to evaluate the relative expression of the genes *FoxP*, *creb, dat *and *TH *in *O. vulgaris*.

**Conclusion:**

We analyzed the expression profiles of some genes here identified for *O. vulgaris *by applying RT-qPCR analysis for the first time in cephalopods. We validated candidate reference genes and found the expression of *ubi *and *tubA *to be the most appropriate to evaluate the expression of target genes in the brain of different octopuses. Our results also underline the importance of choosing a proper normalization strategy when analyzing gene expression by qPCR taking into appropriate account the experimental setting and variability of the sample of animals (and tissues), thus providing a set of HGK which expression appears to be unaffected by the experimental factor(s).

## Background

Relative quantification of mRNA transcripts requires endogenous normalizers, *i.e. *reference genes. The normalization of the expression of target genes favors the elimination of unspecific variation caused by differences in starting material, RNA extraction, enzymatic efficiencies, transcriptional activity and presence of inhibitors in the samples. A suitable reference gene has to *i*. be adequately expressed in the tissue of interest, *ii*. not be co-regulated, and *iii*. show comparable expression levels with target genes. In addition, it should show reduced variability in expression levels among samples and experimental conditions [1-4].

A brief review of the literature shows that a limited number of genes is commonly utilized in RT-qPCR experiments as reference. However, strategies have been developed to validate the use of reference genes experimentally [2,4-10] and to apply computational approaches that allow to monitor the stability of genes in respect to others in all samples [11,12], thus allowing proper normalization of RT-qPCR data.

The aim of the present study is to identify candidate genes in the cephalopod mollusc *Octopus vulgaris *that could be used in RT-qPCR experiments as internal reference (housekeeping genes, HKGs) to normalize the expression of target genes. Such an approach is novel for this taxon and may result of great advantage in future experiments given the complexity of the behavioral repertoire of this animal when compared with its relatively simple neural organization [13,14].

## Results

The brain (supra- and sub-oesophageal masses and optic lobes [15]) and, as control tissue, the mantle of naïve *O. vulgaris *were utilized as samples in this study. Two different sets of individuals were used: a broad collection (for both size and age) caught throughout the year (set a) and a restricted sample (in body size) collected exclusively during the summer (set b).

### Expression stability of candidate reference genes

Likewise other studies, we chose *16S *rRNA (encoded in the mitochondrial DNA), β-actin (*actB*), elongation factor 1A (*EEF1A*) and α-tubulin (*tubA*) as candidate reference genes (sequences available in Gene Bank, Table 1). In addition, partial sequences of *18S *rRNA and ubiquitin (*ubi*) were also identified for this work (see Table 1).

**Table 1:** Gene products, accession number (AN) and gene ontology accession number (GO) of genes considered in this work.

**Gene product**	**AN**	**GO^a^**	**P**	**Primer sequence 5' – 3'**	**AS**	**E^b^**
**α-tubulin**	X15845	0005874	F	ACTGGTGTCCAACTGGCTTC	105	2.00
			R	TGCTTAACATGCACACAGCA		

**β-actin**	AB053937	0005200	F1	TGATGGCCAAGTTATCACCA	103	1.90
			R1	TGGTCTCATGGATACCAGCA		
			F2	TCCAGGCTGTGTTGTCTCTG	148	1.80
			R2	AGATCACGACCAGCCAAGTC		

**CREB**	FJ617443*	0003700	F	ACAGTATGCCCAAGGTCCTG	122	1.80
			R	TTCCAGTGGTTGCCATAACA		

**Dopamine transporter**	FJ617441*	0005329	F	GCCCTAGACGGCATCAAATA	109	2.00
			R	ATCCTGGTCCAAGGGAAAAG		

**Elongation factor**	AY651883	0006414	F	ACGAAGGCTGGGAAATTGA	104	2.00
			R	TGGTCTCTCCGTTGGTCTCT		

**Forkhead box protein P**	FJ617444*	0003700	F	TCCACACCTGCCATGAGTT	149	2.00
			R	GATTGGTCCCACACTGCTG		

**Tyrosine hydroxylase**	FJ617442*	0018336	F	CTCATTGCAGACATGGCATT	128	1.70
			R	GCGTGAGTCGGAAACAGATT		

**Ubiquitin/ribosomal protein S27a**	FJ617440*	0016567	F	TCAAAACCGCCAACTTAACC	113	1.90
			R	CCTTCATTTGGTCCTTCGTC		

**16S**	AJ616309	0005200	F	TTGGGGCTAGAATGAATGGT	112	1.90
			R	GGTCTTTTCGTCCCTTTAAACA		

**18S**	FJ617439*	0005200	F	AGTTCCGACCGTAAACGATG	142	1.80
			R	CCCTTCCGTCAATTCCTTTA		

Primer sequences (F: forward; R: reverse), amplicon size in bp (AS) and calculated reaction efficiencies (E) for RT-qPCR experiments are also reported. Newly identified genes in *O. vulgaris *are marked by an asterisk.

**a**. According to Gene Ontology (, last visited Aug, 2008) for each gene is listed a GO accession number.

**b**. Efficiency for each primer pair was derived from standard curves using five serial dilutions of cDNA samples, as described in Methods.

**Figure 1:**
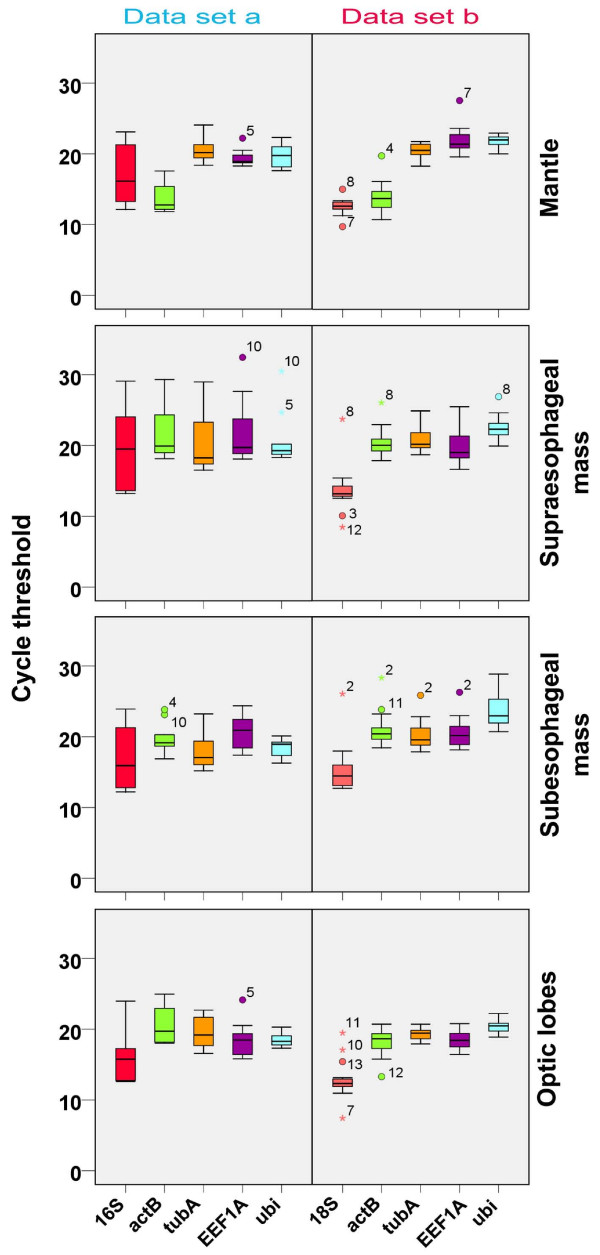
**RT-qPCR cycle threshold (Ct) values of candidate reference genes among different tissues in two data sets**. Cycle threshold distribution values (Ct) of *16S*, *actB*, *tubA*, *EEF1A *and *ubi *(set a, left panel) and *18S*, *actB*, *tubA*, *EEF1A, ubi *(set b, right panel) from mantle, supraoesophageal and suboesophageal masses and optic lobes of *O. vulgaris*. The distribution is shown by vertical box plot as medians (lines), interquartile range (boxes) and ranges (whiskers). Circles mark outliers with values between 1.5 and 3 times the interquartile range; asterisks mark outliers having Ct more than three times the interquartile.

The range of cycle thresholds for the HKG analyzed (Figure 1) resulted quite different among samples. It is noteworthy to report that *16S *rRNA exhibited the highest range of variation in Ct (note the larger interquartile range in Figure 1), when compared with the other genes, in all tissues considered (set a). In addition, as expected for a cytoskeleton structural gene, the expression of *actB *was higher in the mantle than in the brain (set a and b) also when compared with the other candidate reference genes (Figure 1; but note also *18S*).

**Figure 2:**
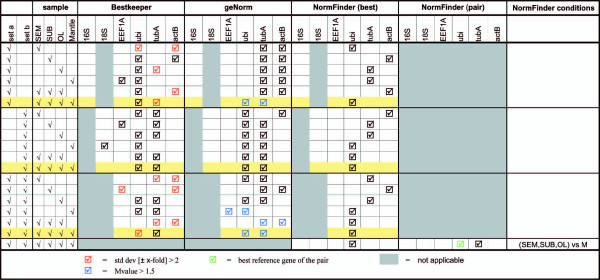
**Searching for the most stable candidate reference genes**. Candidate reference genes for normalization were identified according to BestKeeper [18], geNorm [14] and NormFinder [19] procedures applied to the two set of samples (set a and set b) in *O. vulgaris *mantle (Mantle), supra-oesophageal mass (SEM), sub-oesophageal mass (SUB) and optic lobes (OL). In all tissues, stepwise exclusion was applied for those genes with expression variability above the required values for each algorithm. Values above the recommended thresholds are highlighted. M value (M) is the average pairwise variation of one selected reference gene compared to all the other control genes.

The gene expression stability of the candidate reference genes for our two octopus samples sets was evaluated with BestKeeper [16], geNorm [12] and NormFinder [17]. A tabularized overview of the results is presented in Figure 2. A stepwise elimination of reference genes according to the results obtained from each analysis provided differences and commonalities in the results of the application for the three algorithms. Interestingly, a different result was obtained for each set of samples (set a vs set b) and when the different tissues were considered (Figure 2). When analyzing all tissue samples together for animals belonging to set a (highlighted row), Bestkeeper and NormFinder revealed *ubi *as the best candidate. GeNorm suggested the pair *tubA*-*ubi *to be the best combination of two genes, but with M values above the recommended threshold. In addition, the expression of *tubA *was considered too variable for Bestkeeper.

The analysis of samples taken from a more homogeneous set of animals (set b, highlighted row) provided, as expected, a consensus between the three algorithms. In fact, each software indicated the pair *tubA*-*ubi *as the best combination of reference genes (*ubi *is the best gene according to NormFinder).

A different pattern appeared when both sets of samples were considered (Figure 2). Again, geNorm was not capable to provide a combination of reference genes because the variability in expression resulted in M values above the suggested level of acceptance. Similarly, BestKeeper indicated *tubA *as the best reference gene, but suggested that the levels of *ubi *mRNA in the different samples were too variable to be recommended as reference. NormFinder found *ubi *as the best HKG.

NormFinder also allows to define experimental conditions/samples in a data set [17]. For our purposes, here we considered the mantle as control tissue when studying the expression of different genes in the brain. In these circumstances, NormFinder revealed *ubi *and *tubA *as the best combination of reference genes for evaluating the expression of target genes in animals belonging to different sets.

### Expression level of target genes

**Figure 3:**
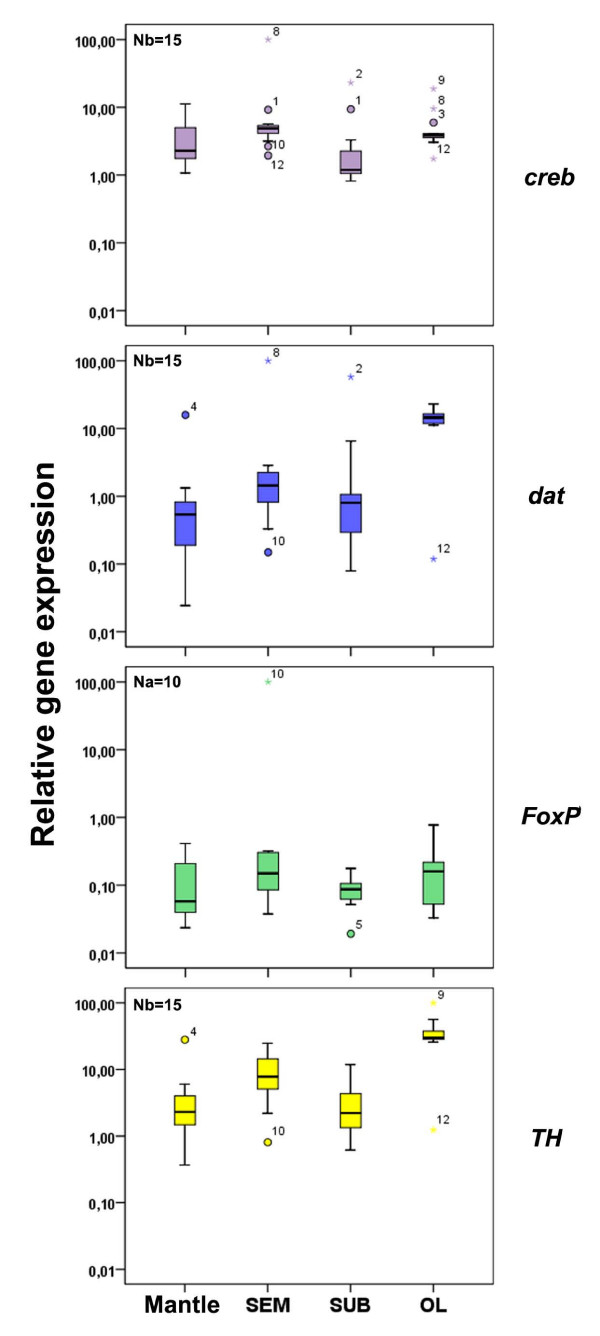
**Relative target gene expression in different tissues for each data set**. Relative expression distribution (box plots: for details see legend to Figure 1) of *creb*, *dat *and *TH *(set b, N = 15) and *FoxP *(set a, N = 10) in *O. vulgaris *mantle, supra-oesophageal mass (SEM), sub-oesophageal mass (SUB) and optic lobes (OL). M value (M) represents a measure of gene-stability and corresponds to the average pairwise variation of one selected reference gene compared to all the others. Relative gene expression (y-axis) was calculated using *tubA *and *ubi *as reference genes; see Methods for details.

In order to apply this pair of reference genes (*tubA*-*ubi*), we analyzed the relative expression of target genes in the two sets of animals in different tissues (Figure 3). The two transcription factors considered in this study [Forkhead box-P (*FoxP*): set a; c-AMP response element binding (*creb*): set b] did not have significantly different levels of expression in different tissues, similar to what resulted by analyzing the expression profile of dopamine transporter (*dat*, set b) in the octopuses (P > 0.05 for all comparisons). On the contrary, tyrosine hydroxylase (*TH*, set b) mRNAs reached expression levels in the optic lobes that were significantly higher when compared with those observed in other tissues (P < 0.05; Figure 3).

## Discussion and conclusion

In this study, we analyzed the expression profiles of some genes here identified for *Octopus vulgaris *for the first time. We applied RT-qPCR analysis, which is unprecedented in cephalopods, and, as a prerequisite, we also validated candidate reference genes. Normalization strategies are required to correct sample-to-sample variability in order to reveal gene-specific variation among experimental conditions and/or tissues. Similarly to what occurs in other animal models [e.g. [18,19]], normalization of mRNA levels to the cell number is not possible for *O. vulgaris *tissues.

Our experiments showed that the expression of several so called housekeeping genes vary among different conditions (possibly body size or seasonality) and/or tissues (i.e. mantle vs brain), but also when the different parts in the brain of the octopus (masses) are considered. However, in such circumstances an algorithm that takes into account different conditions in a given experimental design (NormFinder [17]) results to be beneficial for the identification of suitable reference genes to be used in RT-qPCR experiments. This was our case. NormFinder allowed us to identify *ubi *and *tubA *as the appropriate reference genes for most of the analyzed samples. Moreover, this gene pair resulted to be chosen as the best combination when a tissue (mantle in our condition) is used as control to study the expression of different genes in the brain. We applied this reference gene pair to analyze the level of expression of our target genes (*creb*, *dat*, *TH*, *FoxP*) in different regions of *Octopus *brain applying a geometric approach that take into account primer efficiencies for both reference and target genes.

In our experimental conditions, *16S *and *18S *rRNAs, *EEF1A *and *actB *resulted not reliable as reference genes in terms of stability and relative levels of expression, in analogy to what is reported for other species [e.g. [6,9,10,20]].

In particular, the expression of *16S *rRNA allowed to identify two discrete groups of animals (60-fold differences in Ct; data not shown) in our sample. This did not correlate with sex, seasonality or the body size of the animals and was independent from the tissues considered. The fact that *16S *rRNA is a mitochondrial gene suggests that this may be related with physiological (and/or metabolic) status of the animals. Similarly, we cannot exclude that *18S *rRNA expression may be influenced by physiological conditions. Moreover, we found that *actB *reached higher levels of expression in the mantle when compared to the brain; it also resulted expressed with high interquartile ranges among samples, similarly to what resulted in other experimental settings [21]. Surprisingly, the three algorithms did not indicate *EEF1A *as a suitable reference gene in our experimental conditions (except for Bestkeeper: mantle set a and SUB set b). However, its expression is comparable to the other putative reference genes (Figure 1). This resembles what recently found in other systems [22].

In order to account for the largest inter-individual variability in the octopus, we analyzed some target genes separately in two different groups of animals, one representing the whole population (set a) and one more restricted to sub-adults of the summer season (set b). The expression levels detected for each animal group suggest a correlation between data set heterogeneity and gene expression variability as deduced by the elevated Ct value variability obtained from samples belonging to octopuses of set a.

Moreover, octopuses of our samples showed low expression levels for transcription factors when compared with mRNA coding for proteins highly utilized in the metabolic pathway (i.e. *FoxP *and *creb *vs *dat *and *TH*; data not shown). It is interesting to note that dopamine-related genes were abundantly expressed in the optic lobes of *O. vulgaris*, confirming previously published data [review in [23]].

In conclusion, we were able to identify reference genes to be utilized for normalization in particular conditions in RT-qPCR experiments designed to test gene expression in different tissues of *O. vulgaris *and to describe inter-individual variability in gene expression in naïve octopuses.

## Methods

### Animals

All the octopuses were collected from the wild and maintained in seawater until sacrifice. The animals were anesthetized in a sea water solution using MgCl_2 _[24]. A portion of the mantle (without the skin) and the brain was dissected out the animal. The brain was divided into its parts: supra-oesophageal mass (SEM), sub-oesophageal mass (SUB) and optic lobes (OL). Samples were transferred in plastic tubes (1 ml of Eurozol, Euroclone), immediately frozen in liquid nitrogen, and stored at -80°C until further processed.

#### Set a

The octopuses (*Octopus vulgaris*, N = 10) were collected from different locations of the Bay of Napoli (Italy) throughout the year (2006). Their weight ranged from 30 to 2100 g, spanning across a broad size/age range.

#### Set b

The octopuses (*O. vulgaris*, N = 15) were caught in the Bay of Napoli in the same season (June – July, 2006); their size ranged between 200–550 g.

### RNA extraction and quantification

Total RNA was extracted using Eurozol (Euroclone) according to the manufacturer's instructions. Contaminating DNA was degraded by treating each sample with Turbo DNase Kit (Ambion) according to the instruction manual.

For all RNA samples, the absence of DNA contamination was tested by performing PCR with β-actin primers. The quantity and purity of total RNA extracted was estimated monitoring both the absorbance at 260 nm and ratios 260/280 nm and 260/230 nm by Nanodrop (ND-1000 UV-Vis Spectrophotometer; NanoDrop Technologies). The quality of RNA was evaluated by gel electrophoresis. Intact rRNA subunits (*28S *and *18S*) were observed on the gel indicating minimal degradation of the RNA.

### cDNA synthesis

For each sample, 2 μg of total RNA extracted was retrotranscribed with SuperScript™ First-Strand Synthesis System for RT-PCR (Invitrogen) following the manufacturer's instructions. The cDNA was stored at -20°C until further use. cDNA was diluted 1:100 with H_2_O prior to use in RT-qPCR experiments, and 1:1500 when using *18S *primers.

### Isolation of target and reference genes

Target and reference genes were obtained from *O. vulgaris *using primers designed on conserved regions by means of bioinformatic analysis comparing homologous sequences available in Gene Bank for the different *taxa *(see Additional file 1).

To isolate *FoxP*, retrotranscription reactions were performed using SuperScript™ One-Step RT-PCR Systems (Invitrogen) on total RNA extracted from the brain of adult octopuses using TRIZOL^® ^(Invitrogen) with inosinate degenerate primers (RPPfw 5'-AGACCGCCITTYACITAYGC-3' and AVWrev 5'-TCRTCIACVGTCCAIACIGC-3' for forward and reverse, respectively). Primers WKNfw 5'-TGGAAGAATGCCGTGCGCCA CA-3' and WKNrev 5'-TGTGGCGCACGGCATTCTTCCA-3' were used to obtain the 5' and 3' ends respectively using GeneRacer™ (Invitrogen) following the manifacturer's protocol. At the same time a PCR screening of cDNA libraries from octopus brain were performed to confirm the transcripts obtained (1111 bp).

For all cases (*18S rRNA*, *ubi*, dopamine transporter: *dat*, and tyrosine hydroxylase: *TH*) cDNAs were synthesized using SuperScript™ First-Strand Synthesis System for RT-PCR (Invitrogen) from total RNA extracted from brains of adult octopuses using Eurozol (Invitrogen) according to the manufacturer's instructions. The cDNA obtained was used as template in PCR reactions to amplify genes of interest using primer pairs designed by Primer3 software [25].

For *18S rRNA*, a 442 bp fragment was amplified with primers designed on the basis of *Eledone cirrhosa 18S rRNA *sequence (GeneBank accession number: AY557467): 18Sfw 5'-CGTTTTCCTCGATCAAGAGC-3' and 18Srev 5'-CGAACTCGCGAAAGAAGAAG-3'.

For ubiquitin (*ubi*), a 324 bp cDNA sequence coding for ubi/ribosomal S27a protein was identified using the following primers: UBfw 5'-TGTCAAGGCAAAGATTCAAGA-3' and Ubrev 5'-GGCCATAAACACACCAGCTC-3'. These primers were designed on the basis of sequences present in *Octopus *eye EST library (, last visited: Aug, 2008).

For dopamine transporter (*dat*) we analyzed the ortholog sequence alignment of vertebrates and invertebrates. Degenerated oligonucleotides were designed to amplify conserved regions. A 506 bp fragment coding for *dat *was amplified using the following primers: DATfor1 5'-TCKGGIAARGTDGTBTGGTT-3' and DATrev3 5'-ATIGCYCIGADCCNCCRAA-3'.

For tyrosine hydroxylase (*TH*), the most conserved regions of vertebrate and invertebrate ortholog sequences were identified and degenerated oligos were designed for PCR reactions. The primers Thfor1 5'-RTSTTYCAGWSYACICAGTA-3' and Threv2 5'-AAYTCVACRGTGAACCAGTA-3' were used to amplify a fragment of 539 bp.

cDNA amplified fragments were purified from gel agarose using QIAquick^® ^Gel extraction kit (Qiagen) and cloned into pCRII-TOPO vector (Invitrogen) according to the instruction manual. The resulting plasmids were sequenced using M13 reverse primer and T7. Sequences of the PCR products obtained were analyzed by BLASTX and BLASTP programs.

The c-AMP response element binding protein (*creb*) cDNA sequence was found after screening *O. vulgaris *cDNA library constructed from mRNA of the supra-oesophageal mass. The cDNA sequence coding for *Aplysia creb1α *was used to screen the library (gift from Dr E.R. Kandel Laboratory). The sequence identified was 4313 bp long and coded for a protein of 296 amminoacids.

The resulting alignments of *O. vulgaris *translated sequences with invertebrate and vertebrate orthologues are summarized in Additional file 1.

### Primer design for RT-qPCR

All RT-qPCR primers were designed by Primer 3 software [25]. Table 1 lists the primers' sequences together with amplicon size. Target genes' sequences amplified by the primer pairs were evaluated with MFOLD software [26] in order to check for secondary structures at the site of primer binding. Specificity of PCR products was checked by melting curve analysis followed by gel electrophoresis and DNA sequencing.

### Real Time PCR

The efficiency of each primer pair (Table 1) was calculated according to standard methods curves using the equation E = 10^-1/slope^. Five serial dilutions were set up to determine Ct values and reaction efficiencies for all primer pairs. Standard curves were generated for each oligonucleotide pairs using the Ct value versus the logarithm of each dilution factor.

Diluted cDNA was used as template in a reaction containing a final concentration of 0.3 μM for each primer and 1× FastStart SYBR Green master mix (total volume of 25 μl). PCR amplifications were performed in a Chromo4™ Real-Time Detector (Biorad) thermal cycler using the following thermal profile: 95°C for 10 min, one cycle for cDNA denaturation; 95°C for 15 sec and 60°C for 1 min, 40 cycles for amplification; 72°C for 5 min, one cycle for final elongation; one cycle for melting curve analysis (from 60°C to 95°C) to verify the presence of a single product. Each assay included a no-template control for each primer pair. To capture intra-assay variability all RT-qPCR reactions were carried out in triplicate (set a) or duplicate (set b). Fluorescence was measured using Opticon Monitor 3.1 (Biorad).

### Stability analysis of reference genes

The distribution of the Ct was first calculated for each tissue considering all the samples and reported as box plot. The Ct values were obtained from the average of each animal tissue.

Three different gene normalization algorithms were utilized in this work: BestKeeper [18], geNorm [14] and NormFinder [17]. For each one a step-wise exclusion method have been applied in order to identify the best candidate reference genes.

### Expression analysis of target genes

The expression of each target gene (relative to the most stable reference genes [11]) was calculated applying the following formula:



were:

*ubi*: ubi

*tubA*: tubulin

*trg*: target gene

### Data analysis

Raw Ct data were exported to a worksheet for further analysis. SPSS 16 was used for statistical analysis (One-way ANOVA followed Dunnett post-hoc test). Significance level was set at 5%.

## Authors' contributions

OS and IZ participated in the design of the study, performed all the experiments and drafted the manuscript. FR and MB carried out data analysis and helped to draft the manuscript. MB participated in the design of the study and assisted in RT-qPCR experiments design and realization. MIA and EB participated in the design and coordination of the study and helped to draft the manuscript. GF conceived and supervised the study. All authors read and approved the final manuscript.

## Supplementary Material

Additional file 1 **The alignment of *O. vulgaris *translated sequences with their invertebrate and vertebrate orthologous**. For each gene alignment, identity matrix and cDNA sequence are provided. Click here for file
