# Supplementary material for: Selection and validation of a set of reliable reference genes for quantitative RT-PCR studies in the brain of the Cephalopod Mollusc Octopus vulgaris
Source: BMC Mol Biol. 2009 Jul 14;10:70. doi: 10.1186/1471-2199-10-70 (PMC2722649; doi:10.1186/1471-2199-10-70)
Supplement: Additional file 1 — The alignment of O. vulgaris translated sequences with their invertebrate and vertebrate orthologous. For each gene alignment, identity matrix and cDNA sequence are provided. [file 1471-2199-10-70-S1.pdf]

c-AMP response element binding protein (CREB)

alignment

```
                            *        20         *        40         *        60         *        80
A.  mellifera    : -MESMVEENGSAVDP----LSAGSQSGD--------------------AAPAIATSVQSVIQPNQQSVIQTATNIQPVAIS :  56
D. melanogaster  : MDNSIVEENGNS--------SAASGSND--------------------VVDVVAQQAAAAVGGGG----------GGGGGG :  43
L. stagnalis     : ----MSAGNGPG--------TADIENG------------------------TRGISVVHVSIPN-----------QAIQVQ :  34
H. sapiens       : ----MTMESGAENQQSGDAAVTEAENQQMTVQAQPQIATLAQVSMPAAHATSSAPTVTLVQLPNG----------QTVQVH :  67
O. vulgaris      : ----MSEVPTSQ--------TSEIENG------------------------SQEISIVHVTIPN-----------QPVQVQ :  34

                           *       100         *       120         *       140         *       160
A.  mellifera    : KGNVILVSKPNSVIQTAQ--ASLQTLQVVETGSDDSFSDSEESPEQRGGILTRRPSYKKILNDLGGGENGRLPPLESSSEC : 135
D. melanogaster  : GGGNPQQQQQNPQSTTAG--GPTGATNNAQGGGVSSVLTTTANCNIQYPIQTLAQHGLQVQSVIQANPSGVIQTAAGTQQQ : 122
L. stagnalis     : P--VIQAN--PTVIHSAGNYQTIQVVRVAAVDE--DLSSDDSDAKRRREILARRPSYRKILNDLSS---PVKMEDDSNS-- : 104
H. sapiens       : G--VIQAA-QPSVIQSPQ-VQTVQISTIAESEDSQESVDSVTDSQKRREILSRRPSYRKILNDLSSDAPGVPRIEEEKSEE : 144
O. vulgaris      : S--VIQSSHQPSVIQTTG--PNVQTIQVATVND--DMT-DGAEGRKRREILARRPSYRKILNELSSECPPIKIEEPTNQDG : 108

                          *       180         *       200         *       220         *       240
A.  mellifera    : DSNVDSEVSS-------------HSLHYQTVIPAGTIQIATQ---GEGVPGLHTLTMSNA-------ATAGG-AIVQYAQG : 192
D. melanogaster  : QQALAAAT----------------AMQKVVYVAKPPNSTVIHTTPGNAVQIPPTFPCKIKPEPNTQHPEDSDESLSDDDSQ : 187
L. stagnalis     : -----------------------SQSQDGEYVQGGTLQLTAHGDATPATQGLQTLTMTNASPTSASNSTGGT-TIVQYAQG : 161
H. sapiens       : ETSAPAITTVTVPTPIYQT----SSGQYIAITQGGAIQLANN--GTDGVQGLQTLTMTNA-----AATQPGT-TILQYAQT : 213
O. vulgaris      : QETTEAASNLSTNSIQYQPATVYSQMEGLPLVPSGAIQITTHPEGTVAGQGLQTLTMTNANPSSGAGTTSGTATIVQYAQG : 189

                         *       260         *       280         *       300         *       320
A.  mellifera    : QDTQ-FFVP--------------------------GHGVVVE--------------DAARKRELRLLKNRQAARECRRKKK : 232
D. melanogaster  : HHRSELTRRPS------------------------YNKIFTEISGPDMSDNSGIAEDQTRKREIRLQKNREAARECRRKKK : 244
L. stagnalis     : PDGQ-FYIP-------VGGTNVQAYQIPGS----LAQGVVMT--GSPISSPQHMSEEGSRKRELRLLKNREAARECRRKKK : 228
H. sapiens       : TDGQQILVPSNQVVVQAASGDVQTYQIRTAPTSTIAPGVVMA---SSPALPTQPAEEAARKREVRLMKNREAARECRRKKK : 291
O. vulgaris      : PDGQ-FLIPVT-----VGGTNVQAYQIATS----LPQGVVMATTGSPITSPQQLSEEAARKRELRLLKNREAASECRRKKK : 260

                        *       340         *       360
A.  mellifera    : EYIKCLENRVAILENRNQTLIEELKSLKQLCEPKTD- : 268
D. melanogaster  : EYIKCLENRVAVLENQNKALIEELKSLKELYCQTKND : 281
L. stagnalis     : EYVKCLENRVAVLENQNKTLIEELKALKELYCQKDA- : 264
H. sapiens       : EYVKCLENRVAVLENQNKTLIEELKALKDLYCHKSD- : 327
O. vulgaris      : EYVKCLENRVSVLENQNKTLIEELKALKELYCQKDG- : 296
```

CREB identity matrix

	**Accession number**	**A. mellifera**	**D. melanogaster**	**L. stagnalis**	**H. sapiens**	**O. vulgaris**
**A. mellifera**	CAD24872	100%				
**D. melanogaster**	NP_001097018	23%	100%			
**L. stagnalis**	BAC20140	35%	23%	100%		
**H. sapiens**	NP_004370	33%	19%	42%	100%	
**O. vulgaris**	ACN38053	34%	21%	59%	43%	100%

O. vulgaris creb cDNA complete sequence (4313bp)

GCGGCGTTGTGCTGTGACGCTGTAGTTGACGTGGCCACTACGTATTCTGCTAATTTATCCACTCCGATTGCGGCAAAG
TTGGTGACGAAAAGTACGTTAACTGGTAAGGAAGAACTTTCATCTACATGACCTTTGACGTTATTACTTTCGATGTTG
GCCAAGATAACAGGTGAATTGTCACCAATACATTCAGTGTGAAATATTCTACAAATATATTCTATTGAGTGAAGATG
CGCATGGGCTCTCAGTTTGTCCCCTTTTCTCTGGATACTGTGTGCATCAGTTGGGTCTAATTTTGAACATGGCCTTCTG
TGCTTATATTTTTTCCAGAGTAGAGAATGTCTGAAGTGCCAACCTCACAGACCAGTGAGATTGAAAACGGCTCGCAG
GAGATATCTATTGTGCATGTGACAATACCTAACCAACCAGTCCAAGTGCAATCTGTTATACAATCTAGCCATCAACCA
TCAGTGATACAGACAACTGGCCCAAATGTTCAGACTATTCAGGTAGCTACAGTAAATGATGATATGACAGATGGTGC
TGAAGGAAGAAAGAGGAGAGAGATATTAGCTAGAAGACCTTCATATCGTAAAATTTTGAATGAATTATCGTCCGAGT
GTCCACCTATCAAAATTGAGGAACCTACAAATCAAGACGGTCAAGAGACCACTGAAGCAGCTTCAAACCTCAGTACA
AATTCCATACAGTACCAGCCTGCAACAGTATACAGCCAGATGGAAGGTTTGCCATTAGTCCCATCTGGTGCCATCCA
AATAACTACACATCCAGAAGGAACAGTGGCTGGTCAAGGTCTTCAAACGTTAACAATGACCAATGCAAATCCATCAT
CTGGTGCTGGTACAACATCTGGTACTGCCACTATTGTACAGTATGCCCAAGGTCCTGATGGACAATTTTTAATTCCTG
TTACAGTTGGTGGCACAAATGTGCAGGCTTACCAGATTGCAACATCTTTGCCTCAAGGTGTTGTTATGGCAACCACTG
GAAGTCCAATTACCAGTCCTCAACAATTATCAGAAGAAGCAGCTCGGAAGCGAGAACTTCGTCTATTAAAAAACAGA
GAGGCAGCTTCAGAATGTCGACGGAAGAAGAAAGAATATGTGAAATGCCTGGAAAATCGAGTATCAGTTCTGGAAA
ATCAAAATAAAACATTAATAGAGGAATTGAAGGCATTAAAAGAATTGTATTGTCAAAAAGATGGATAAATGGATTTT
GGCGATTATGTTCTCCCTAAATATGAAGAGGCAACACAGCAGTTTGCTGGTCTCACTCTTCGAACAATTTGGAGTTTA
ACTTTTATTTCAACCACAGTTTACTACAAACAATTGCATCTTCAAATCATTTCAAAATAATAAATACAAATAAAGTTT
TTTGTTTTTTTTTTGTTTTTGTTCTGCTTTATTTTCCTAAGTCTAAATTTTTTTCTTTTCTTTTATTTTTAATAGTTTTCTTT
GGAAAGTTTTTATTTGAATAATACCGCCCCCACTAAATATTTAAAAAAATCATATTTTTAGGCTGACATATTTTAAAT
GACTGTGAAGGGCTGGGTGTGGCAACTTAATGSAATATCATTATTTTCTCTGTTGTGGAGATTTTTAATTTTCTATAAA
TTCAATGGAAATTCTTAAAATATTTATTCTCTCATCATCTGTTATTTTTTATTCCCTTTTGTTGCACACCTTTTTGTTTTC
CCCATCTTTTTGATTTTTTTAAAAGAATTTTTGTTTAATATTTGAAGAATGATAGAAATTTGTTTTTAAATTACTTTTTG
TTGTAATTTTGTTTCATTTTCTTTGAATATTATTAATAGTCCAATTCCAAAAGAAAAGAATGCTCTTAGGTGGAGGGG
AAAATGAGGTAGAGTGGTGAGAACAAATTTGGGGTAATATCAGAAAAAAAAAACAACAAAAAAAAAAAACAAAGAAA
AAGGGAAAAAGACTTTTAATAAAACTGGGATGTCTTGCTATGTAATTTCATTTGGAACTTAACCATAACTATTTTCAA
CTTTTACAATTCCAAATTGAAAGTATCTCATTTTGAACTGGTGTTCATTCAGTTTAACAGATTGTTCTCTGAAACTGAT
ATGAAATTACTTGGGTGGAAATTCTGCAGACCTTTTTACTTCATGCATTTTGTCTGTTTCTATAAAATTGTTTACATTTT
CAAATAGCTTTTCTTTCTTTCTTACTGTTATTTTTTTCTTCTTTCATTTAGAAAATTGTATTTTATTTTCTTGCAAGTTAC
TTTTAATTGTTCTATTTTGGTAAATATAAAGAGGTTAAAGAATATACATTAATTTTTCAAATTTCCCCATTCCATTGTC
TACATATTAAACTGAAGTTTGTTGTTAATAAGCTCTTGTTCAGTCATGGACAACCTGTGACCTTCAGGACTTTCTGAA
TAGCATGCCAACAAAAAATAACCTTGAATTTCTTTTAGTGTGCAGTCCCTCTAAAAATGAGCTGTATCTCATGTGCCC
TGCTTCTCAAGAAAGGTTGCCTATGTCTGACCAAGTAACTAAACTCATAGTTAGATCTAGCAGATTGAAGTTCTTCTA
ACTTCATAATAGTCAATGAATTTTTTCAAAATTGGGCAGAAAACCAATTCTGAAGATAAACTATTTTTAACTTTAGGA
TAATATCAGCTTTAAAATTTTACATTTTGTTATATCTTGATGATATTTGATGAATGTTCCATGAGATACTGTTAGCTTT
TGGGGATCACAGATTTAGCATATCACAACAACTGTTGTAACGTTTCCTAATCCATAGTTCATCTGAGCCCGGAGACAA
CGCTCGGTTGTTATAGGTTTTAGGGCTATACTGAAGCTGGTCTTGAACAGGCTGTTTAATCAATGTTAAACTGTACAT
GAAGTGACTTGTATATAAGCCTGCAAGACAATTACACTAATATTATACTAATGAATGTTAAAAGCAATAAACTTATG
TGAAACATTACCGTTTAATTAATTCACAAACTAAATGAATACAGGTGAATATCCTAAATCAAATCACACCAAGGTCA
GTTTTATCTTTCATCTTTTTGGAGTTGATTAATAAAGGATGAGCAGTAGGAATTGACACTGCTCTTCCATTCTCTTTCA
AGAACTGGTTGTGTCTGTGCTGTGAGAAGGACATGGGAGATCACTTCCACTGTACCTTCTTTCAGTTTATCATAGAGA
AAGAGTGTGGTTACCACAGCTCTGGTAGAATATGGCATATGATGATGGCTAGAATCCTAAATAAGATTTGTATGTGT
CATCTAATTTCATGCCAGCTTAGAAAAAAAAAGGCATCAAATGATGATCATAATAAATAGTTTATTGGTTGAGCAAAA
TGTTTGTCTTACATTATTAGGGTCATGAATAACATGGACTGAAGTATTTTAATAAACCTTATTCACACCATTCCATTCA
TTCATATACAGCATTTAAAAAAAAAAATTCAGGTATGTATTTGAGTTTGTTGGCTTTTATGTGTTTCAGAACCTGATCA
AGACCAATTGAATATTTTACTATCACGGCTTTTGCCTATAAATGATCACTGTATCCTGGAATTAATTGTATAGTGCTTT
TTTTTTATGTGTTGGCTACTATGAGAACTATGGTAATACTACTACTACTACTACTAATAATAATTTTGTCTAATCCCAT
TGTAATCATGACTTTACATTATCTTCCTTGTTTATTGTTTTTAAATACCTTTAGTAGTAGTTTTTGTCTACTAATTGGAT
GGATGACCCCAGTTACTCATTGTTTTTCAACTTCATGTATTTTACTATACATGATATTTTCCTGGGGTTCTTGCCCTCA
ACTTTATTGTTGGTATTCATAATTTGCATCACTACATTCCCTCTGTGTGTATGTGTGTGTGCATGTGTGTTCACTTTTCA
GTCTTGCTAACAAGAACGTAGAATATACTTCATTGACAAAGAATGAATCTTGACTATTACTTCACCAGCTGGCCAGAT
TTATTCCCAACTTCACTGACTTTATTTTCCAAATATTTCAATTACCTGATTGTATCTCTTGCTTGTTGTTTTTAGATTTT
AAATTCCTCTTGTGGGAGTTTTTGTCTAATAATTGCATAGATAATCTCAGTTTACATTAGGTTGGGGTGGGGGAGGGA
CACTTTATGTAGCATATTCCACCTTCATATTACTGCTTTTTTGCTAATTGCTTTCAAATTTTAAAATATTACACTATTTG
AAAGACAATGTGTTGAAGTGAATTATTCATTTTTCCCCCTTTCCCTGAGCAAATAAAACAAAACAACAAAAAAAAAAA
AAAAAAAAA

Dopamine transporter (DAT)

alignment

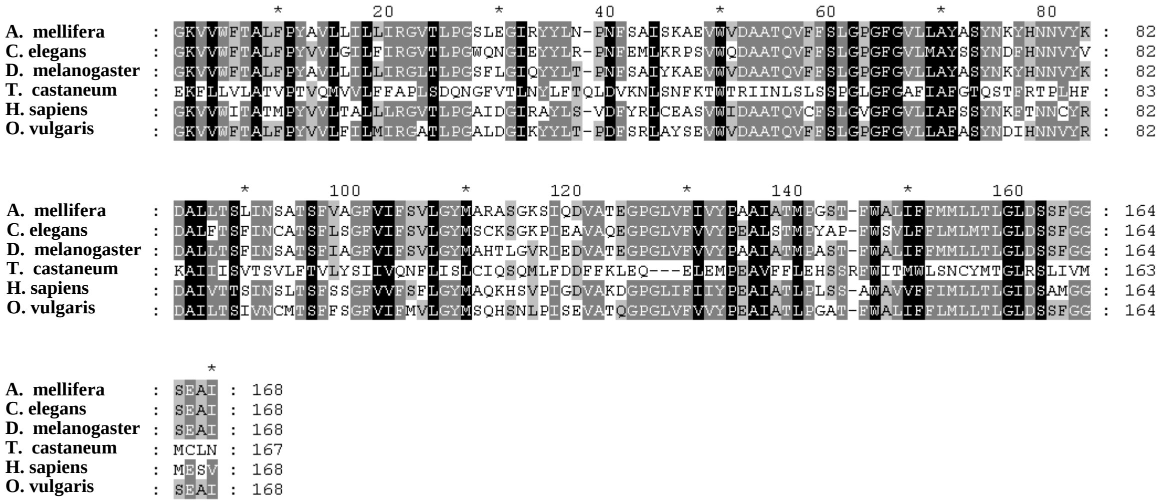

DAT identity matrix

	Accession number	A. mellifera	C. elegans	D. melanogaster	T. castaneum	H. sapiens	O. vulgaris
**A. mellifera**	XP_624619	100%					
**C. elegans**	Q03614	73%	100%				
**D. melanogaster**	AAF76882	90%	73%	100%			
**T. castaneum**	XP_972640	15%	17%	15%	100%		
**H. sapiens**	Q01959	61%	60%	60%	21%	100%	
**O. vulgaris**	ACN38051	76%	73%	75%	18%	63%	100%

O. vulgaris dat cDNA partial sequence (506bp)

CTGGGAAGGTTGTGTGGTTTACTGCCTTATTCCCATACGTTGTCCTGTTCATTCTTATGATTCGTGGGGCAACACTGCCGGGGGCCC
TAGACGGCATCAAATATTATCTGACACCAGACTTCTCAAGACTTGCATATTCCGAGGTGTGGGTGGATGCGGCCACACAAGTCTTC
TTTTCCCTTGGACCAGGATTCGGTGTTTTATTGGCATTTGCAAGTTATAATGATATTCATAATAATGTTTATCGAGACGCCATACTA
ACCAGTATCGTTAACTGTATGACGAGTTTTTTCTCTGGATTTGTTATCTTCATGGTTTTGGGTTATATGTCACAACACTCAAATCTAC
CCATTTCAGAGGTGGCTACACAAGGACCTGGTTTAGTGTTCGTTGTATATCCAGAAGCCATAGCTACCCTCCCAGGTGCAACATTT
TGGGCATTGATATTCTTCCTCATGTTGCTTACCCTAGGTCTAGACAGTTCGTTTGGTGGCTCAGAAGCCATC

Forkhead Box - P family - protein (FOXP)

alignment

```
                           *        20         *        40         *        60         *        80
A. mellifera   : --NVPKSVAGLNVSSRRSDMNGTTPLDEKPLDVSSNDKVHPLYGHGVCKWPGCEVICEDYQAFLKHLNTEHTLDDRSTAQA :  79
C. elegans     : SVMALITHLQHEHPPSDKSNEEMRNQIEKVESIEHKLSVERSRLQGMMQHLRMKPSPDTTTPNLVKMEAQSPLRSPKIEGA :  81
D. melanogaster: -LMFSDAERFLRSKDNEVCNNDFSYMHD---EFAMRKYYHPLFAHGICRWPGCEMDLEDITSFVKHLNTEHGLDDRSTAQA :  77
S. purpuratus  : -MMNGLLDGSHIHPGFLLAHQGMMMPHEDRLANPNN---HPLYAHGMCKWPGCETVCEDFPAFLKHLSTEHALDDRSTAQA :  77
T. castaneum   : --LNDGSPGLLGVATNRPSRD-EAPLEDKLERTTTPDRVHHLYGHGVCKWPGCEIICEDLQAFIKHLNTEHTLDDRSTAQA :  78
H. sapiens     : SSNTSKASPPITHHSIVNGQSSVLSARRDSSSHEETGASHTLYGHGVCKWPGCESICEDFGQFLKHLNNEHALDDRSTAQC :  81
O. vulgaris    : -LLSGGLGGSLLNQSAVSIKQ----------EQEASSPSHPLFRHGVCKWPGCDTPNEDFPSFIKHLNSEHQLDDRSTAQA :  70

                         *       100         *       120         *       140         *       160
A. mellifera   : RVQMQVVSQLEIQLQKE---RDRLTAMMHHLHVAK------------QMASPEPIECLSGTTLDR--KLSSEKKQSIPNMS : 143
C. elegans     : AFSIQPAQQFQQQTTSQPQVSPTSEAASSLLSIAATVAASTAAAVTSPINQISTVPSVSSMPSFLNHQLSTSSQPSSQQAS : 162
D. melanogaster: RVQMQVVSQLESHLQKE---RDRLQAMMHHLYLSK------------QLLSPTKIDRKDVPGREG--KFCR-SPLTVNSIG : 140
S. purpuratus  : RVQMQVVNQLEIQLTKE---RERLAAMMAHLHMKPPID---------SKQETPKSSVQSSPSLSKPVSVVQHTAPSLPMVP : 146
T. castaneum   : RVQMQVVSQLELQLQKE---RDRLQAMMHHLHLSK------------QLSSPEPHKDGS-VVTPT--KMAVNTSLSQPSVS : 141
H. sapiens     : RVQMQVVQQLEIQLSKE---RERLQAMMTHLHMRP-SE---------PKPSPKPLNLVSSVTMSK--NMLETSPQSLPQTP : 147
O. vulgaris    : RVQMQVVSQLEIQLIRE---KDLLQAMMQHLHMKPQVDKSDIILPVLPKQQPHQQQQTQQALQQAHMDITSQLPSIPPLS : 148

                        *        180         *       200         *       220         *       240
A. mellifera   : NMSGMPPLPN------------------MPGSMPTMPTMPS--------MAG--PIRRRISDKSALSLAG----------- : 185
C. elegans     : GSSGPLLQRA------------------ASSASTETSPNPD--------SKSFVPRRSRISDKTVQPIAT----------- : 206
D. melanogaster: -----RPIRQ------------------TNSPSPLNLPMVN--------STNLCSIKKRNHDKNTFSINGGLPYMLERAGL : 190
S. purpuratus  : SSTPPVPITS------------------IASA-PYHLTSHH--------VTPGGPIRRRNSEKYGLSLST----------- : 189
T. castaneum   : ----IGPMVS------------------AVRSPVLHSPAAN--------VAG--PIRRRLNDKSALSLAGGLPYMLERAGL : 190
H. sapiens     : -TTPTAPVTP------------------ITQG-PSVITPAS--------VPNVGAIRRRHSDKYNIPMSS----------- : 189
O. vulgaris    : NASNVPLLTSTPAMSLSPTTTCTSPQMVISTAHPAHSPVQQSPQHQQQASSSVGPIRRRVSDKCNLPISAGMQRDMENAFL : 229

                       *        260         *       280         *       300         *       320
A. mellifera   : ----EIQRNREFYKNADVRPPFTYASLIRQSIIESPDKQLTLNEIYNWFQNTFCYFRRNAATWKNAVRHNLSLHKCFMRVE : 262
C. elegans     : ----DIAKNRDFYRTNDVRPPYTYASLIRQAIMESSDCQLTLNEIYTWFTETFAYFRRNAATWKNAVRHNLSLHKCFQRVE : 283
D. melanogaster: DVQQEIHRNREFYKNADVRPPFTYASLIRQAIIDSPDKQLTLNEIYNWFQNTFCYFRRNAATWKNAVRHNLSLHKCFMRVE : 271
S. purpuratus  : ----EIHHNSDFYKNTDVRPPFTYAALIRQGIIDAPDRQLTLNEIYNWFTRTFAYFRRNAATWKNAVRHNLSLHKCFVRVE : 266
T. castaneum   : DVQQEIQRNREFYKNADVRPPFTYASLIRQSIIESPDKQLTLNEIYNWFQNTFCYFRRNAATWKNAVRHNLSLHKCFMRVE : 271
H. sapiens     : ----EIAPNYEFYKNADVRPPFTYATLIRQAIMESSDRQLTLNEIYSWFTRTFAYFRRNAATWKNAVRHNLSLHKCFVRVE : 266
O. vulgaris    : S-FVEIQRNREFYRTTDVRPPFTYASLIRQAIIESPHRQLTLSEIYQWFANTFAYFRRNEATWKNAVRHNLSLHKCFMRVE : 309

                      *        340         *       360         *       380         *       400
A. mellifera   : N-VKGAVWTVDEVEFYKRRPQRACSTTGGVPSKSPTLTHSPTMYGDALNANLQAALGDSNMGFLNNSMCTSTTTSPDKEHV : 342
C. elegans     : QNVKGAVWTVDDSEFYRRRPNRAS-----------------------------ATRSQPQTPLPDDISQQKLFDTSALSSF : 335
D. melanogaster: N-VKGAVWTVDEIEFYKRRPQRT-----------------------------AGIGNN---------LTGATNSPDTNYF : 312
S. purpuratus  : N-VKGAVWTVDEIEFMKRRPQRMGSLNTPPSTPNTPTTPISKSVDMLPTEDMPLNLETHMHPMGGSLPLLSDAAAAAADHA : 346
T. castaneum   : N-VKGAVWTVDEVEFYKRRPQRS-----------------------------AGLGD----------------------- : 298
H. sapiens     : N-VKGAVWTVDEVEYQKRRSQKIT--GSPTLVKNIPT---SLGYGAALNASLQAALAESSLPLLSNPGLINNASSGLLQAV : 341
O. vulgaris    : N-VKGAVWTVDEVEFYKRRPQKL-------------------------------------------------SGYVFFL : 338

A. mellifera   : LA : 344
C. elegans     : LE : 337
D. melanogaster: VA : 314
S. purpuratus  : IQ : 348
T. castaneum   : -- :   -
H. sapiens     : HE : 343
O. vulgaris    : FA : 340
```

FOXP identity matrix

	Accession number	A. mellifera	C. elegans	D. melanogaster	S. purpuratus	T. castaneum	H. sapiens	O. vulgaris
**A. mellifera**	XP_391970	100%						
**C. elegans**	NP_001023148	29%	100%					
**D. melanogaster**	AAY51579	48%	27%	100%				
**S. purpuratus**	XP_782985	47%	29%	41%	100%			
**T. castaneum**	XP_969436	57%	28%	59%	43%	100%		
**H. sapiens**	EAL24367	50%	29%	40%	50%	43%	100%	
**O. vulgaris**	ACN38054	40%	29%	42%	40%	45%	39%	100%

O. vulgaris FoxP cDNA partial sequence (1111bp)

TGCAAGTGGCCGGGGTGCAGGCTGTTATCAGGTGGTCTTGGTGGTAGTCTATTGAATCAGTCGGCTGTTAGCATTAAAC
AGGAGCAGGAAGCGTCTAGTCCATCGCACCCACTTTTCCGACATGGTGTTTGTAARTGGCCGGGGTGTGATACGCCAA
ATGAAGACTTTCCTTCCTTCATTAAGCATCTGAACTCTGAGCACCAGTTAGATGACAGAAGTACAGCACAAGCAAGAG
TACAAATGCAGGTTGTTAGTCAACTTGAAATACAGCTTATACGGGAAAAAGATCTTCTGCAAGCAATGATGCAGCACT
TACACATGAAACCTCAAGTAGATAAATCAGATATTATTCTCCCAGTACTTCCAAAGCAGCAGCCGCACCAGCAGCAGC
AGCAGACCCAACAAGCTTTGCAACAGGCCCACATGGATATTACATCTCAACTCCCTTCAATTCCTCCATTGTCCAATGC
TTCAAATGTCCCCCTTTTAACCTCCACACCTGCCATGAGTTTATCACCCACTACTACCTGTACATCACCTCAAATGGTCA
TCTCTACTGCCCATCCTGCACATTCACCTGTCCAGCAGTCACCCCAGCACCAACAGCAAGCCTCCAGCAGTGTGGGAC
CAATCCGTCGACGGGTATCAGACAAGTGCAATCTGCCTATCTCTGCTGGAATGCAGAGAGACATGGAGAACGCATTTC
TTTCTTTTGTAGAAATCCAGAGAAATCGCGAATTTTACCGAACAACAGACGTCMGACCRCCRTTCACRTATGCCTCTCT
CATTCGTCAGGCCATCATAGAATCTCCACATCGACAATTAACATTGAGTGAAATCTATCAGTGGTTCGCAAACACATTT
GCTTATTTCCGGCGGAATGAAGCTACGTGGAAGAATGCCGTGCGCCACAACCTTAGTCTTCACAAATGTTTTATGCGAG
TTGAGAATGTTAAGGGGGCTGTATGGACCGTCGACGAGGTAGAGTTYTACAAACGGAGACCACAAAAACTGAGTGGG
TATGTTTTTTTTTTGTTTGCTTGATTGTTGTCATTTTTTTTTTTTACAAAACTTTACGAAAAGCAAAAACTCACAAAAAAA
AAAAAAAAAA

Tyrosine hydroxylase (TH)

alignment

```
                                 *        20         *        40         *        60         *        80
A. mellifera    : FADKEYRARRKFIAEIAFAYRYGDAIPTVPYTETETETWTRVFNTLVDLVPKHACAEYRRNFKKMQEEKIFEPHRIPQLQEVS :  83
C. elegens      : HGDVAYIARRKFLNDQALEFKFGDEIGYVDYTEEEHATWKAVYEKLGDLHLSHTCAVYRQNLKILQEEKVLTADRIPQIRDVN :  83
D. melanogaster : FADKVYRQRRKEIAEIAFAYKYGDPIPFIDYSDVEVKTWRSVFKTVQDLAPKHACAEYRAAFQKLQDEQIFVETRLPQLQEMS :  83
S. purpuratus   : FTDKDYRERRQRIADVAFKYKHGQPIPRVEYTDDELRTWGLIYRQLKALFPTHACKEHIDAFNILEKEGLYSESFIPQHEDVS :  83
T. castaneum    : FADKEYRARRKEIAEIAFAYKYGDPIPYIQYTETETKTWGSVFNTVLELMPKHACSEYCRVFKMLQEEDIFTPDRIPQLEEMS :  83
H. sapiens      : FSDQVYRQRRKLIAEIAFQYRHGDPIPRVEYTAEEIATWKEVYTTLKGLYATHACGEHLEAFALLERFSGYREDNIPQLEDVS :  83
O. vulgaris     : FADKEYRRRRKLIADMAFTYRHNKSIPRVEYSEDEIKTWKHVYVNIKNLFPTHACSKHRHFFDLLEKKGIYSENFIPQLEDVS :  83

                       *        100         *       120         *       140         *       160
A. mellifera    : EFLKKNTGFTLRPAAGLLTSRDFLSSLAFRVFQSTQYIRHIKSPYHTPEPDCIHELLGHMPLLADPSFAQFSQEIGLASLGAS : 166
C. elegens      : KFLQKKTGFELRPCSGLLSARDFLASLAFRVFQTTTYLRHHKSPHHSPEPDLIHELLGHVPMFSDPLLAQMSQDIGLMSLGAS : 166
D. melanogaster : DFLRKNTGFSLRPAAGLLTARDFLASLAFRIFQSTQYVRHVNSPYHTPEPDSIHELLGHMPLLADPSFAQFSQEIGLASLGAS : 166
S. purpuratus   : NFLKGKTGFQLRPVAGLLSARDFLASLAFRVFQATQYVRHSSAPMHTPEPDCCHELLGHVPMLADPTFAQFSQEIGLASLGVA : 166
T. castaneum    : NFLKRHTGFSLRPAAGLLTARDFLASLAFRIFQSTQYVRHKNTPYHTPEPDCIHELLGHMPLLADPSFAQFSQEIGLASLGAS : 166
H. sapiens      : RFLKERTGFQLRPVAGLLSARDFLASLAFRVFQCTQYIRHASSPMHSPEPDCCHELLGHVPMLADRTFAQFSQDIGLASLGAS : 166
O. vulgaris     : NFLKQQTGFQLRPVAGLLSARDFLSSLAFRVFQCTQYIRHGSKPDHTPEPDCVHELLGHVPMLADPEFAEFSQELGLASIGTS : 166

                     *
A. mellifera    : DEEIEKLSTIYWF : 179
C. elegens      : DEHIEKLSTVYWF : 179
D. melanogaster : DEEIEKLSTVYWF : 179
S. purpuratus   : DEDITRLATLYWF : 179
T. castaneum    : DAEIEKLSTVYWF : 179
H. sapiens      : DEEIEKLSTLYWF : 179
O. vulgaris     : DEDIEKFATLYWF : 179
```

TH identity matrix

	Accession number	A. mellifera	C. elegans	D. melanogaster	S. purpuratus	T. castaneum	H. sapiens	O. vulgaris
**A. mellifera**	NP_001011633	100%						
**C. elegans**	AAV58890	59%	100%					
**D. melanogaster**	CAA53802	77%	55%	100%				
**S. purpuratus**	XP_001177854	59%	52%	59%	100%			
**T. castaneum**	NP_001092299	79%	56%	81%	61%	100%		
**H. sapiens**	AAI04968	64%	57%	63%	70%	63%	100%	
**O. vulgaris**	ACN38052	62%	50%	58%	71%	60%	70%	100%

O. vulgaris TH cDNA partial sequence (539bp)

TTCGCGGACAAAGAATACAGAAGAAGAAGGAAGCTCATTGCAGACATGGCATTCACATACAGGCATAACAAGAGTATTCCCAGAGTT
GAATATTCGGAAGATGAGATAAAGACTTGGAAACATGTTTATGTGAACATAAAAAATCTGTTTCCGACTCACGCCTGCTCAAAGCACC
GCCATTTCTTTGATTTACTGGAAAAGAAAGGAATCTACAGTGAAAACTTTATACCACAGCTTGAAGATGTTTCCAATTTCCTGAAACAA
CAAACAGGTTTCCAACTGCGCCCGGTAGCTGGACTGTTATCAGCACGTGACTTCTTGTCCAGCTTAGCATTTCGTGTTTTCCAATGTACT
CAGTACATACGCCATGGCTCCAAACCGGACCACACACCAGAACCTGATTGTGTGCATGAACTGCTAGGACATGTCCCAATGCTGGCAG
ATCCTGAATTCGCTGAGTTTTCCCAAGAACTTGGTTTGGCTTCAATAGGGACCTCCGATGAAGATATTGAAAAGTTTGCAACGCTTTAC
TGGTTCAC

Ubiquitin/ribosomal protein S27a (UBI)

alignment

```
                          *        20         *        40         *        60         *        80
A. mellifera    : VKAKIQDKEGIPPGQQRLIFAGKQLEDGRTLSDYNIQKESTLHLVLRLRGGAKKRKKKNYSTPKKIKHKKKKVKLAVLKFYKV : 83
D. melanogaster : VKAKIQDKEGIPPDQQRLIFAGKQLEDGRTLSDYNIQKESTLHLVLRLRGGAKKRKKKNYSTPKKIKHKRKKVKLAVLKYYKV : 83
S. purpuratus   : VKAKIQDKEGIPPDQQRLIFAGKQLEDGRTLSDYNIQKESTLHLVLRLRGGAKKRKKKNYTTPKKTKHKKKKVKLAVLKFYKV : 83
T. castaneum    : VKAKIQDKEGIPPDQQRLIFAGKQLEDGRTLSDYNIQKESTLHLVLRLRGGAKKRKKKNYSTPKKIKHKKKKVKLAVLKFYKV : 83
H. sapiens      : VKAKIQDKEGIPPDQQRLIFAGKQLEDGRTLSDYNIQKESTLHLVLRLRGGAKKRKKKSYTTPKKNKHKRKKVKLAVLKYYKV : 83
O. vulgaris     : VKAKIQDKEGIPPDQQRLIFAGKQLEDGRTLSDYNIQKESTLHLVLRLRGGAKKRKKKNYTTPKKNKHKKKKVKLAVLKYYKV : 83

                        *        100
A. mellifera    : DENGKIHRLRRECPMEQCGAGVFMA : 108
D. melanogaster : DENGKIHRLRRECPGENCGAGVFMA : 108
S. purpuratus   : DENGKITRLRRECPSEECGAGVFMA : 108
T. castaneum    : DENGKIHRLRRECPGEQCGAGVFMA : 108
H. sapiens      : DENGKISRLRRECPSDECGAGVFMA : 108
O. vulgaris     : DENGKITRLRRECPNEECGAGVFMA : 108
```

UBI identity matrix

	**Accession number**	**A. mellifera**	**D. melanogaster**	**S. purpuratus**	**T. castaneum**	**H. sapiens**	**O. vulgaris**
**A. mellifera**	XP_397323	100%					
**D. melanogaster**	NP_476778	95%	100%				
**S. purpuratus**	XP_784247	94%	93%	100%			
**T. castaneum**	XP_969023	98%	97%	95%	100%		
**H. sapiens**	CAA44911	90%	93%	94%	91%	100%	
**O. vulgaris**	ACN38050	93%	94%	97%	94%	95%	100%

O. vulgaris ubi cDNA partial sequence (324bp)

TGTCAAGGCAAAGATTCAAGATAAGGAGGGAATTCCTCCAGATCAGCAAAGATTGATCTTTGCTGGTAAGCAGCTTGAGGAT
GGCCGTACACTGTCAGACTACAATATTCAAAAGGAATCAACCCTTCATTTGGTCCTTCGTCTGCGAGGTGGCGCCAAGAAGCG
CAAGAAGAAGAATTACACCACTCCCAAAAAGAATAAGCATAAGAAGAAGAAGGTTAAGTTGGCGGTTTTGAAATATTACAAG
GTTGATGAAAATGGTAAAATCACTCGTTTGCGTCGTGAGTGTCCCAACGAGGAATGTGGAGCTGGTGTGTTTATGGC

18S ribosomal RNA (18S rRNA)

alignment

```
                              *        20         *        40         *        60         *        80
A. mellifera   : GTTTTCATTAATCAAGAACGAAAGTTAGAGGTTCGAAGGCGATCAGATACCGCCCTAGTTCTAACCATAAACGATGCCAGC :  81
C. elegans     : GTCTTCATTAATCAAGAACGAAAGTCAGAGGTTCGAAGGCGATTAGATACCGCCCTAGTTCTGACCGTAAACGATGCCATC :  81
E. cirrhosa    : GTTTTCCTCGATCAAGAGCGAAAGTCGGAGGTTCGAAGACGATCAGATACCGTCGTAGTTCCGACCGTAAACGATGCCGAC :  81
S. purpuratus  : TTTTTCATTAATCAAGAACGAAAGTTAGAGGTTCGAAGGCGATCAGATACCGCCCTAGTTCTAACCATAAACGATGCCGAC :  81
H. sapiens     : GTTTTCATTAATCAAGAACGAAAGTCGGAGGTTCGAAGACGATCAGATACCGTCGTAGTTCCGACCATAAACGATGCCGAC :  81
O. vulgaris    : GTTTTCCTCGATCAAGAGCGAAAGTCGGAGGTTCGAAGACGATCAGATACCGTCGTAGTTCCGACCGTAAACGATGCCGAC :  81

                            *       100         *       120         *       140         *       160
A. mellifera   : CAGCGATCCGCCGAAGTT-CCT-CCGATGACTCGGCGGGCAGCTTCCG--GGAAACCAAAGCTTTTGGGTTCCGGGGGAAG : 158
C. elegans     : TCGCGATTCGGAGGTTT--------TTGCCCTGCCGAGGAGCTATCC--GGAAACGAAAGTCTTTCGGTTCCGGGGGTAG : 152
E. cirrhosa    : TGGCGCTCCGCCGGCGTTCGCT-TCGATGACCCGGCGGGGAGCCTGCGCGGGAAACCATAGTC----GGTTCCGGGGGGAG : 157
S. purpuratus  : TGACGATCCGCCGGCGTT-ACT-CCCATGACGCGGCGG-CAGTCTAAG--GGAAACCAAAGTCTTTGGGTTCCGGGGGAAG : 157
H. sapiens     : CGGCGATGCGGCGGCGTT-ATT-CCCATGACCCGCCGGGCAGCTTCCG--GGAAACCAAAGTCTTTGGGTTCCGGGGGGAG : 158
O. vulgaris    : TGGCGCTCCGCCGGCGTTCGCT-TCGATGACCCGGCGGGGAGCCTGCGCGGGAAACCATAGTC----GGTTCCGGGGGGAG : 157

                          *       180         *       200         *       220         *       240
A. mellifera   : TATGGTTGCAAAGCTGAAACTTAAAGGAATTGACGGAAGGGCACCACCAGGAGTGGAGCCTGCGGCTTAATTTGACTCAAC : 239
C. elegans     : TATGGTTGCAAAGCTGAAACTTAAAGAAATTGACGGAAGGGCACCACAAGGCGTGGAGCTTGCGGCTTAATTTGACTCAAC : 233
E. cirrhosa    : TATGGTTGCAAAACAGAAACTTAAAGGAATTGACGGAAGGGCACCACCAGGAGTGGAGCCTGCGGCTTAATTTGACTCAAC : 238
S. purpuratus  : TATGGTTGCAAAGCTGAAACTTAAAGGAATTGACGGAAGGGCACCACCAGGAGTGGAGCCTGCG-CTTAATTTGACTCAAC : 237
H. sapiens     : TATGGTTGCAAAGCTGAAACTTAAAGGAATTGACGGAAGGGCACCACCAGGAGTGGAGCCTGCGGCTTAATTTGACTCAAC : 239
O. vulgaris    : TATGGTTGCAAAACAGAAACTTAAAGGAATTGACGGAAGGGCACCACCAGGAGTGGAGCCTGCGGCTTAATTTGACCCAAC : 238

                         *       260         *       280         *       300         *       320
A. mellifera   : ACGGGAAACCTCACCAGGCCCGGACACCGGAAGGATTGACAGATTGATAGCTCTTTCTTGATTCGGTGGGTGGTGGTGCAT : 320
C. elegans     : ACGGGAAAACTCACCCGGTCCGGACACCATTAGGACTGACAGATTGAAAGCTCTTTCTCGATTTGGTGGTTGGTGGTGCAT : 314
E. cirrhosa    : ACGGGAAAACTCACCCGGTCCGGACACCGGTAGGATTGACAGATCGATAGCTCTTTCTCGATTCGGTGGGTGGTGGTGCAT : 319
S. purpuratus  : ACGGGAAAACTCACCCGGCCCGGACACAGTGAGGATTGACAGATTGAGAGCTCTTTCTTGATTCTGTGGGTGGTGGTGCAT : 318
H. sapiens     : ACGGGAAACCTCACCCGGCCCGGACACGGACAGGATTGACAGATTGATAGCTCTTTCTCGATTCCGTGGGTGGTGGTGCAT : 320
O. vulgaris    : ACGGGAAAACTCACCCGGTCCGGACACCGGTAGGATTGACAGATCGATAGCTCTTTCTCGATTCGGTGGGTGGTGGTGCAT : 319

                       *       340         *       360         *       380         *       400
A. mellifera   : GGCCGTTCTTAGTTGGTGGAGCGATTTGTCTGGTTAATTCCGATAACGAACGAGACTCTAGCCTGCTAAATAGACGTAACT : 401
C. elegans     : GGCCGTTCTTAGTTGGTGGAGTGATTTGTCTGGTTTATTCCGATAACGAGCGAGACTCTAGCCTGCTAAATAGTTGGCGAA : 395
E. cirrhosa    : GGCCGTTCTTAGTTGGTGGAGCGATCCGTCTGGTTGATTCCGATAACGAACGAGACTCCGGCCTGCTAACTAGCGCGTCGA : 400
S. purpuratus  : GGCCGTTCTTAGTTGGTGGAGCGATTTGTCTGGTTAATTCCGATAACGAACGAGACTCTGGCTTGCTAAATAGTTGCGCCA : 399
H. sapiens     : GGCCGTTCTTAGTTGGTGGAGCGATTTGTCTGGTTAATTCCGATAACGAACGAGACTCTGGCATGCTAACTAGTTACGCGA : 401
O. vulgaris    : GGCCGTTCTTAGTTGGTGGAGCGATCCGTCTGGTTGATTCCGATAACGAACGAGACTCCGGCCTGCTAACTAGCGCGTCGA : 400

                     *       420
A. mellifera   : TATGGTATCTCGAAGGCCCTC : 422
C. elegans     : TCTTC---------------- : 400
E. cirrhosa    : TTAGTGCTTTTCCGGTAGCGT : 421
S. purpuratus  : CCCC----------------- : 403
H. sapiens     : CCCCCGA-------------- : 408
O. vulgaris    : TTAGTGCTTTTCCGGTAGCGT : 421
```

18S rRNA identity matrix

	**Accession number**	**A. mellifera**	**C. elegans**	**E. cirrhosa**	**S. purpuratus**	**H. sapiens**	**O. vulgaris**
**A. mellifera**	AY703484	100%					
**C. elegans**	EU196001	84%	100%				
**E. cirrhosa**	AY557467	81%	77%	100%			
**S. purpuratus**	L28055	90%	85%	81%	100%		
**H. sapiens**	NR_003286	90%	86%	83%	92%	100%	
**O. vulgaris**	FJ 617439	81%	76%	99%	80%	83%	100%

O. vulgaris 18S rRNA (422bp)

CGTTTTCCTCGATCAAGAGCGAAAGTCGGAGGTTCGAAGACGATCAGATACCGTCGTAGTTCCGACCGTAAACGATGCCGAC
TGGCGCTCCGCCGGCGTTCGCTTCGATGACCCGGCGGGGAGCCTGCGCGGGAAACCATAGTCGGTTCCGGGGGGAGTATGGT
TGCAAAACAGAAACTTAAAGGAATTGACGGAAGGGCACCACCAGGAGTGGAGCCTGCGGCTTAATTTGACCCAACACGGGA
AAACTCACCCGGTCCGGACACCGGTAGGATTGACAGATCGATAGCTCTTTCTCGATTCGGTGGGTGGTGGTGCATGGCCGTTC
TTAGTTGGTGGAGCGATCCGTCTGGTTGATTCCGATAACGAACGAGACTCCGGCCTGCTAACTAGCGCGTCGATTAGTGCTTT
TCCGGTAGCGT
